# Comparison of Fluorescent Probes for IDH-Wildtype Glioblastoma, Metastatic Brain Tumors, and PCNSL: A Biomechanical Perspective

**DOI:** 10.3390/ijms27104495

**Published:** 2026-05-17

**Authors:** Zelong Zheng, Ami Kobayashi, Yosuke Kitagawa

**Affiliations:** 1 Department of Neurology, Brigham and Women’s Hospital, Harvard Medical School, Boston, MA 02114, USA; eydengzelong@scut.edu.cn; 2 Department of Neurosurgery, Guangzhou First People’s Hospital, School of Medicine, South China University of Technology, Guangzhou 510180, China; 3 Department of Medical Sciences and Innovation, Institute of Medical Research, Tohoku University, Sendai 980-8574, Japan; 4 Department of Neurosurgery, Graduate School of Medicine, The University of Tokyo, Bunkyo-ku, Tokyo 113-8655, Japan

**Keywords:** fluorescent probes, IDH-wildtype glioblastoma, metastatic brain tumors, primary central nervous system lymphoma, resection

## Abstract

Intraoperative fluorescence-guided surgery is an important adjunct to brain tumor resection. However, fluorescent probe performance varies across molecularly and histopathologically distinct entities, including IDH-wildtype glioblastoma, metastatic brain tumors (MBTs), and primary central nervous system lymphoma (PCNSL), and the mechanisms underlying this variability remain poorly understood. We propose a mechanistic framework integrating biomechanical constraints, molecular barrier heterogeneity, and probe-specific pharmacokinetics to explain cross-tumor differences in fluorescence signal. Probe performance is conceptualized through three sequential bottlenecks: extravasation (blood–brain barrier/blood–tumor barrier permeability and transcytosis), interstitial penetration (extracellular matrix density and hydraulic resistance), and retention/clearance (efflux transporters and metabolic processing). An overlying optical layer, including tissue absorption, scattering, and autofluorescence, further modulates the detected signal. Tumor-specific molecular heterogeneity critically shapes these processes. In IDH-wildtype glioblastoma and legacy high-grade glioma cohorts, heterogeneous expression of ATP-binding cassette transporters has been associated with reduced intracellular accumulation of protoporphyrin IX after 5-aminolevulinic acid administration and may contribute to false-negative fluorescence in selected tumor regions. In MBTs, stage-dependent blood–tumor barrier integrity and vascular programs influence probe delivery, whereas in PCNSL, corticosteroid-sensitive restoration of endothelial barrier function may compromise the performance of leakage-dependent tracers. Together, this framework highlights how tumor biology, barrier function, and probe pharmacology jointly shape fluorescence contrast. Rational probe selection informed by tumor-specific transport and barrier constraints may improve intraoperative visualization of brain tumors and optimize surgical decision-making.

## 1. Introduction

In accordance with the 2021 WHO Classification of Tumors of the Central Nervous System [1], the term “glioblastoma” is used here to refer to glioblastoma, IDH-wildtype, CNS WHO grade 4. Historical studies using “GBM” or “malignant glioma” terminology are interpreted cautiously, because many were conducted before routine IDH-based molecular classification. When IDH status was unavailable, such studies are treated as legacy glioblastoma or molecularly unclassified high-grade glioma evidence rather than as direct evidence for the current WHO-defined entity.

The fundamental surgical objective in brain tumor management varies substantially across tumor entities, and these differences impose distinct demands on intraoperative visualization technologies. In glioblastoma, IDH-wildtype, CNS WHO grade 4 (hereafter, IDH-wildtype glioblastoma), the extent of resection correlates with overall survival, motivating the goal of maximal safe resection, removing as much enhancing and infiltrative tumor as possible while preserving eloquent brain function [2,3,4,5]. The highly infiltrative nature of glioblastoma, however, renders the true tumor margin histologically indistinct, making precise intraoperative delineation challenging [2,5]. In contrast, metastatic brain tumors (MBTs) are typically nodular and well circumscribed, allowing clearer anatomical boundaries during resection. Nevertheless, multiplicity is common, and small satellite metastases may escape detection under conventional white-light microscopy [6,7]. In MBTs, fluorescence guidance has been investigated as a potential adjunct for intraoperative lesion visualization, but reliable detection of occult satellite metastases and consistent improvements in the surgical extent of resection remain unestablished. Primary central nervous system lymphoma (PCNSL) represents a fundamentally different therapeutic paradigm. Surgical resection confers no survival benefit, and the principal role of surgery is largely limited to diagnostic biopsy [8,9]. Fluorescence has therefore been explored primarily as a means to improve targeting of viable tumor tissue and reduce the risk of non-representative sampling, although the utility of fluorescence guidance may be significantly compromised by prior corticosteroid exposure [9,10].

Despite their distinct surgical objectives, these three entities often share a common radiological feature: all typically present with gadolinium enhancement on magnetic resonance imaging (MRI), indicating some degree of blood–brain barrier (BBB) or blood–tumor barrier (BTB) disruption [11]. However, this shared enhancement phenotype does not imply comparable probe delivery, retention, or fluorescence output across tumor entities.

Existing reviews of intraoperative fluorescent probes have largely organized discussion around molecular targets or emission wavelengths, providing comprehensive catalogs of available agents but offering limited mechanistic insight into why probe performance varies across tumor entities [12,13,14,15,16]. For example, 5-Aminolevulinic acid (5-ALA) produces robust fluorescence in many IDH-wildtype glioblastomas and historically defined glioblastoma/GBM cohorts, yet demonstrates inconsistent performance in MBTs and limited utility in PCNSL [2,17,18]. Such discrepancies cannot be fully explained by differences in target abundance or metabolic pathway activity alone, suggesting that additional biological constraints, particularly those governing probe transport, tissue penetration, and retention, play a critical role in determining fluorescence output [19,20]. In particular, prior syntheses have given limited attention to the molecular architecture underlying these barriers, including the enzymes, transporters, and structural proteins that collectively regulate probe access to tumor tissue and subsequent signal generation.

To address this gap, we propose a mechanistic framework that links tissue biomechanics and molecular transport constraints to intraoperative fluorescence. In this model, probe physicochemistry, route of administration, and tumor-specific barrier status jointly shape delivery efficiency, interstitial penetration, and intratumoral retention. Specifically, extracellular matrix (ECM) remodeling, tight-junction regulation, efflux transporter activity, and metabolic enzyme expression interact with probe-intrinsic properties to determine whether a signal can be generated and retained. Beyond these biological factors, optical properties of the tissue environment, including absorption, scattering, and autofluorescence, further shape the detected signal intensity and spatial accuracy.

Using this framework, we define three sequential bottlenecks governing fluorescence signal generation: extravasation, interstitial penetration, and retention/clearance, and examine how each stage is modulated by tumor-specific molecular programs and probe-intrinsic physicochemical properties. The framework is then applied to three clinically important tumor entities (IDH-wildtype glioblastoma, MBTs, PCNSL), highlighting the barrier features that are most likely to dominate probe performance in each context. Finally, we evaluate major probe classes through this unified lens and propose practical recommendations for aligning probe selection with tumor biology, surgical objectives, and underlying molecular and biophysical constraints.

## 2. Scope and Evidence Interpretation

This article was designed as a narrative mechanistic review rather than a formal systematic review or meta-analysis. Targeted searches were performed in PubMed and Web of Science for English-language literature published from January 2005 through December 2025, supplemented by citation tracking from key clinical, translational, and mechanistic studies. The primary scope was fluorescence-guided surgery in IDH-wildtype glioblastoma, metastatic brain tumors, and primary CNS lymphoma, with selected contextual references included when directly relevant to fluorescence mechanisms or historical glioblastoma terminology. Detailed search terms, eligibility considerations, exclusion principles, and evidence categorization procedures are provided in Supplementary Materials, Section S1.

Because the evidence base differs substantially across probe classes, we interpreted key claims according to the type of supporting evidence, including randomized controlled trials, prospective cohorts, retrospective series, and preclinical data only. These categories are used to distinguish clinically established approaches from context-dependent adjuncts and investigational platforms. The resulting evidence categories for key claims are summarized in Table 1.

## 3. Biomechanics-to-Fluorescence Framework

**Figure 1:**
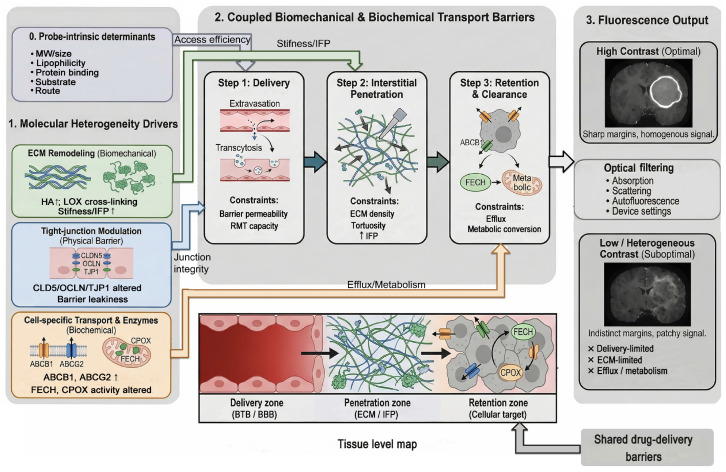
Conceptual framework linking molecular heterogeneity to intraoperative fluorescence via coupled biomechanical and biochemical transport barriers. Created in BioRender. Zheng, Z. (2026) https://BioRender.com/89yred6 (accessed on 13 May 2026).

Detectable fluorescence in tumor tissue emerges from a multistep process shaped by both probe-intrinsic properties and tumor- and measurement-related constraints (Figure 1). At the first level, probe-intrinsic physicochemical properties, including molecular weight, lipophilicity, plasma protein binding, transporter substrate status, and route of administration, determine whether and how efficiently the agent can access the tumor compartment. Once systemic delivery is initiated, three sequential tumor-side bottlenecks govern the fate of the probe: (1) extravasation across the BBB/BTB, (2) interstitial penetration through the tumor ECM, and (3) cellular retention versus clearance. Finally, optical and measurement factors, including tissue absorption, scattering, autofluorescence, and device-specific parameters, transform the underlying biological probe distribution into the fluorescence signal ultimately observed by the surgeon. Importantly, these constraints are not unique to imaging agents. Many of the biological and physical barriers that regulate fluorescent probe delivery also govern therapeutic drug distribution within brain tumors, suggesting that advances in one domain may inform progress in the other. The framework should therefore be interpreted as a mechanistic synthesis of plausible and partially validated constraints, rather than as a set of uniformly proven causal pathways. The strength of evidence varies across links: for example, optical attenuation and probe pharmacokinetics are directly measurable intraoperatively, whereas molecular determinants such as transporter expression, tight-junction remodeling, or ECM composition are often inferred from associative clinical observations or preclinical models.

Probe-intrinsic properties influence access to the tumor compartment. After systemic delivery, three tumor-side bottlenecks shape probe fate: BBB/BTB access, ECM penetration, and cellular retention versus clearance. Optical and measurement factors then filter the biological probe distribution into the signal observed intraoperatively. The same barrier classes also constrain therapeutic drug delivery. Created with Biorender.com.

### 3.1. Extravasation: Delivery Across the Blood–Brain Barrier and Blood–Tumor Barrier

For most systemically administered fluorescent probes discussed in this review, the first major bottleneck is extravasation from the intravascular compartment into the tumor interstitium. In the healthy central nervous system, the BBB tightly restricts both paracellular and transcellular transport. Endothelial tight-junction complexes, including claudin-5, occludin, zonula occludens-1 (ZO-1), seal intercellular clefts, while vesicular transcytosis is minimal under physiological conditions [59,60,61,62]. In brain tumors, however, tumor-associated neovascularization partially disrupts this architecture, generating a blood–tumor barrier (BTB), which exhibits spatially heterogeneous permeability [20,63,64,65]. Downregulation or altered localization of tight-junction proteins, influenced by factors such as vascular endothelial growth factor (VEGF), hypoxia, and inflammatory cytokines, has been associated with increased paracellular permeability and may facilitate passive leakage of small-molecule and protein-bound probes in regions of BTB disruption. This passive paracellular route is likely to represent a major delivery pathway for low-molecular-weight agents such as fluorescein sodium. Large albumin-bound dyes, including indocyanine green (ICG), may also enter tumor tissue in regions of marked BTB disruption through fenestrated or transcellular routes.

Beyond passive leakage, active transcellular transport mechanisms merit consideration, particularly for nanoparticle and macromolecular probes. Caveolae-mediated transcytosis, regulated by caveolin-1 and associated lipid rafts, provides a vesicular pathway for albumin and certain ligand-conjugated carriers [66]. In addition, receptor-mediated transcytosis (RMT) provides an additional mechanism for crossing the endothelial barrier. Receptors such as low-density lipoprotein receptor-related protein 1 (LRP1), transferrin receptor, and insulin receptor are expressed on brain endothelium and can shuttle appropriately engineered nanoparticles across the endothelial monolayer [66,67,68]. Notably, RMT efficiency depends on receptor density, ligand affinity, and competition with endogenous substrates. These factors vary substantially between tumor types and even within individual lesions [69]. Although transcytosis-based delivery strategies may help overcome some limitations of passive permeability, their clinical translation for fluorescence-guided surgery remains at an early stage [70,71].

### 3.2. Interstitial Penetration: Movement Within the Tumor Interstitium

Following extravasation, fluorescent probes must traverse the tumor interstitium to reach target cells distant from the perivascular space. This stage of probe transport is governed by interstitial hydraulic conductivity, diffusion coefficients, and convective flow dynamics, all of which are strongly influenced by the composition and structural organization of the ECM [72]. Consequently, the physicochemical properties of the probe interact with tumor-specific ECM remodeling to determine the spatial distribution of fluorescence signal within tumor tissue.

High-grade gliomas and several metastatic brain tumors exhibit substantial deposition of hyaluronan (hyaluronic acid, HA). The polyanionic and highly hydrophilic structure of HA binds water and generates viscoelastic resistance to interstitial flow, thereby increasing tissue viscosity and reducing the effective diffusivity of macromolecular probes [33,34]. At the same time, collagen, particularly types I and IV, undergoes enzymatic cross-linking mediated by lysyl oxidase (LOX) family enzymes, leading to matrix stiffening and reduced interstitial pore size [35]. These structural alterations, combined with impaired interstitial fluid drainage within tumors, decrease hydraulic conductivity and elevate interstitial fluid pressure (IFP). Elevated IFP, in turn, reduces convective transport and biases probe distribution toward perivascular regions [36]. A practical consequence of these biomechanical constraints is that fluorescence often appears rim-dominant, reflecting areas of greater vascular access, while more central or infiltrative tumor regions may remain relatively under-labeled. Thus, limited interstitial penetration represents a critical bottleneck that can decouple probe delivery from effective tumor-wide fluorescence labeling.

### 3.3. Retention and Clearance: Why Signal Persists or Disappears

Once probes reach tumor cells, the persistence of intracellular fluorescence depends on the dynamic balance between probe accumulation, sequestration, active efflux, and metabolic degradation. Among the most important regulators of intracellular probe retention are ATP-binding cassette (ABC) transporters, particularly ABCG2 (breast cancer resistance protein) and P-glycoprotein (ABCB1). These transporters are expressed in both tumor cells and tumor-associated endothelium and actively export a wide range of substrates, including PpIX, the fluorescent metabolite generated following administration of 5-ALA [29,30,31,73]. Elevated ABCG2 expression has been associated with reduced intracellular PpIX accumulation and diminished fluorescence signal and may contribute to false-negative fluorescence in histologically viable tumor tissue [30,32]. Importantly, transporter expression varies substantially both between tumor types and within individual lesions, introducing an additional layer of molecular heterogeneity that can influence fluorescence detectability [31].

Retention should also be interpreted in a cell-type-specific manner. Persistent fluorescence is not necessarily equivalent to retention within neoplastic cells. Tumor-associated macrophages, resident microglia, and inflammatory or phagocytic cells in peritumoral or treatment-related tissue can internalize fluorescent dyes, nanoparticles, activatable probe products, or fluorescent cellular debris. This is particularly relevant at infiltrative or inflamed tumor margins, where immune and reactive glial cells can generate a signal that overlaps spatially with residual tumor. Thus, the retention/clearance bottleneck includes both tumor cell retention and non-neoplastic cellular sequestration, the latter of which can produce a non-specific background signal and complicate interpretation of tumor boundaries.

Conversely, certain probes exhibit prolonged intracellular retention through lysosomal trapping. Weakly basic or cationic fluorophores can accumulate within acidic lysosomal compartments, where protonation limits their diffusion across lysosomal membranes [74]. This mechanism likely contributes to the prolonged intracellular persistence observed with certain activatable probes and nanoparticle-encapsulated dyes. The interplay between efflux and trapping ultimately defines the practical imaging window. Probes with rapid clearance require precisely timed imaging to capture optimal signal, whereas probes with prolonged retention provide greater intraoperative flexibility but may also accumulate in non-tumor cells, potentially reducing specificity [75].

### 3.4. Optical and Measurement Considerations

The fluorescence signal observed intraoperatively does not directly reflect probe distribution alone but instead represents the result of complex interactions between biological probe localization and optical detection constraints. Factors such as blood absorption, tissue scattering, necrosis, autofluorescence, and device-dependent signal processing can significantly distort fluorescence intensity and apparent tumor margins. To improve cross-study comparability, a minimal set of acquisition and analysis parameters should be systematically reported. These include: (i) the imaging system and optical filter configuration, (ii) excitation power and camera gain or exposure settings, (iii) display thresholding parameters and any quantitative calibration procedures, (iv) timing of imaging relative to probe administration (including any re-dosing), and (v) the contrast metric used for analysis (e.g., tumor-to-background ratio or signal-to-background ratio) along with the method used for its calculation. Without standardized reporting of these parameters, apparent differences in probe performance across studies, tumor types, or patient populations remain difficult to interpret reliably [55,56,57]. Recognition of these optical and measurement-layer constraints is therefore essential for accurate assessment of fluorescence-guided surgery technologies. These optical confounders should be considered at the time each probe class is interpreted, rather than treated only as post hoc limitations. For example, blood products can attenuate blue-light excitation and visible-wavelength emission, necrotic tissue can produce a misleading background signal, and device thresholding can convert a continuous intensity gradient into an apparently discrete margin. Consequently, apparent absence of fluorescence may reflect optical attenuation rather than failed delivery, whereas apparent positive fluorescence may reflect non-specific pooling or background rather than viable tumor.

Although the three-bottleneck framework is intended primarily as a mechanistic synthesis, several of its components can be mapped onto measurable clinical or translational readouts. At present, these parameters should not be interpreted as validated decision thresholds, because quantitative cutoffs have not been standardized across tumor entities, imaging platforms, probe classes, or institutions. Nevertheless, they can be used as practical surrogates to structure preoperative planning and intraoperative interpretation. For example, contrast enhancement and dynamic contrast-enhanced magnetic resonance imaging (DCE-MRI) parameters, including the volume transfer constant (Ktrans), extravascular extracellular volume fraction (Ve), and plasma volume fraction (Vp), can provide indirect estimates of BBB/BTB permeability. Perfusion-derived measures, such as relative cerebral blood volume (rCBV) and relative cerebral blood flow (rCBF), may provide complementary information about vascularity and delivery likelihood for leakage-dependent probes. Magnetic resonance elastography (MRE)-derived tissue stiffness, diffusion-weighted imaging/apparent diffusion coefficient (ADC), edema patterns, and extracellular matrix markers may provide complementary information about tissue stiffness, cellularity, and interstitial transport constraints. During surgery, quantitative fluorescence metrics, including tumor-to-background ratio and signal-to-background ratio, together with kinetic parameters such as wash-in slope, time-to-peak, and wash-out rate, can help distinguish delivery failure, poor penetration, rapid clearance, and optical attenuation when device settings are standardized. Accordingly, the framework can be operationalized as a decision-support tool rather than as a rigid algorithm. A highly enhancing, permeability-dominant lesion may be more compatible with leakage-based probes such as fluorescein or ICG-based approaches, whereas a non-enhancing or steroid-exposed lesion should raise concern for delivery-limited false negatives. Tumors with dense extracellular matrix, restricted diffusion, high cellularity, or suspected elevated interstitial resistance may be less favorable for large, targeted constructs or nanoparticles. Conversely, fluorescence loss at the margin after 5-ALA should be interpreted in light of tumor cellularity, metabolic state, potential efflux, and optical confounders rather than assumed to indicate the absence of tumor.

The framework described above links the physical constraints emphasized in this review, tissue stiffness, barrier permeability, hydraulic resistance/interstitial fluid pressure (IFP), and retention–efflux balance, to specific molecular programs that vary across tumor entities and molecular subtypes.

## 4. Tumor Microenvironment Profiles

We therefore apply the three-bottleneck model (extravasation, interstitial penetration, and retention/clearance) to three clinically important tumor entities, IDH-wildtype glioblastoma, MBTs, and PCNSL, to identify the barrier features most likely to shape probe performance in each context (Table 2).

### 4.1. IDH-Wildtype Glioblastoma and Historically Defined Cohorts

IDH-wildtype glioblastoma is characterized by pronounced spatial heterogeneity in barrier integrity, vascular phenotype, extracellular matrix organization, and treatment-related remodeling. However, many studies historically labeled as “GBM” or “malignant glioma” were conducted before routine IDH-based classification and may therefore represent molecularly heterogeneous legacy high-grade glioma cohorts rather than exclusively the current WHO-defined glioblastoma entity. This distinction is important because IDH-wildtype glioblastoma and astrocytoma, IDH-mutant, CNS WHO grade 4 differ in molecular landscape, microenvironmental organization, vascular behavior, and potentially fluorescence characteristics. Consequently, mechanistic and clinical inferences from older “GBM” studies should be interpreted cautiously when applied to present-day IDH-wildtype glioblastoma.

IDH-wildtype glioblastoma and legacy glioblastoma/GBM cohorts exhibit pronounced spatial heterogeneity in both barrier integrity and microenvironmental composition. The contrast-enhancing tumor core generally corresponds to substantial BBB disruption (Section 3.1). In these regions, leakage-dependent probes such as fluorescein and ICG are more likely to accumulate, and delivery of metabolic probes such as 5-ALA may also be facilitated. However, the infiltrative tumor margin, where isolated tumor cells migrate along white-matter tracts and perivascular spaces, often retains a relatively intact BBB. This preserved barrier limits probe extravasation and contributes to false-negative fluorescence signals at the resection edge [1,20].

Angiogenic signaling pathways are strongly activated in IDH-wildtype glioblastoma, with vascular endothelial growth factor (VEGF) and angiopoietin-2 promoting vascular permeability and abnormal neovascularization [1,79]. At the molecular level, expression of efflux transporters such as ABCG2 and ABCB1 varies across transcriptional subtypes and may be further modulated by prior therapies; these patterns could influence intracellular PpIX retention [73,80], although direct clinical linkage to intraoperative fluorescence intensity remains incompletely established. Additional complexity arises from the presence of necrotic cores, a hallmark of IDH-wildtype glioblastoma pathology. Accumulation of probes within necrotic debris may generate non-specific fluorescence signals, while central hypoxia can alter heme pathway metabolism and compromise metabolic probe retention. Furthermore, reduced perfusion in necrotic regions may further complicate probe penetration and distribution within the tumor mass (Section 3.2 and Section 3.3) [81,82].

### 4.2. Metastatic Brain Tumors (MBTs)

In contrast to the diffuse infiltration characteristic of IDH-wildtype glioblastoma, MBTs are characterized by nodular architecture with relatively well-circumscribed boundaries, facilitating gross-total resection when lesions are surgically accessible. In many cases, the peritumoral zone is dominated by vasogenic edema rather than infiltrative tumor cells, a distinction with practical importance for margin definition [83,84]. However, substantial heterogeneity exists across metastases depending on the primary tumor origin. For example, melanoma metastases are often highly vascularized and demonstrate robust radiographic enhancement, whereas certain adenocarcinoma metastases present with less pronounced disruption of the BTB [85,86].

Barrier properties are also influenced by lesion stage. Macrometastases with established neovasculature often display increased BTB permeability, permitting accumulation of both small-molecule and protein-bound tracers [84]. In contrast, micrometastases, consisting of small clusters of tumor cells at early colonization stages, may remain protected by a relatively intact BBB. This preserved barrier significantly limits the delivery of systemically administered probes and thereby contributes to the difficulty of detecting early metastatic lesions intraoperatively [20,87]. This stage-dependent barrier status has direct implications for detection sensitivity and underscores the need for probes capable of transcytosis or active targeting.

Molecular subtypes further stratify MBT environments beyond the primary site of origin. In particular, angiogenic metastases often develop a greater disruption of BTB and edema, conditions that favor the accumulation of leakage-dependent probes. Conversely, vessel co-option-dominant lesions, in which tumor cells grow along existing host vasculature without inducing substantial angiogenesis, may preserve relatively intact BBB microvasculature and thereby limit delivery despite high tumor cell target expression. In breast cancer metastases, for example, HER2-positive and triple-negative subtypes show distinct vascular signaling and barrier phenotypes, suggesting that molecular subtype-specific assessment of permeability and interstitial transport constraints may ultimately help refine probe selection. This consideration is particularly relevant when interpreting false-negative fluorescence in early micrometastatic disease [83,85].

### 4.3. Primary CNS Lymphoma (PCNSL)

PCNSL presents a unique microenvironmental architecture characterized by an angiocentric growth pattern, in which lymphoma cells infiltrate along perivascular spaces and form concentric cuffs around small vessels. This architecture introduces a distinct constraint at the post-extravasation stage: even when probes successfully exit the vasculature, their distribution may remain biased toward perivascular compartments and may not readily access deeper parenchymal regions.

PCNSL lesions can show variable radiographic enhancement, and early lesions can retain relatively preserved barrier integrity, increasing the likelihood of poor performance by leakage-dependent probes [88,89]. PCNSL is characterized by dense cellularity and a prominent reticulin fiber scaffold composed largely of type III collagen, contrasting with the necrotic and edematous regions typical of IDH-wildtype glioblastoma [88]. This dense matrix architecture may impede interstitial penetration of larger probes while simultaneously promoting retention of certain smaller molecules within tightly packed cellular compartments, representing a distinct constraint at the interstitial penetration stage (Section 3.2).

A clinically critical feature of PCNSL is its marked sensitivity to corticosteroids. Administration of corticosteroids such as dexamethasone prior to biopsy can rapidly reduce contrast enhancement and lead to the so-called “vanishing tumor” phenomenon. Mechanistically, corticosteroids can restore endothelial barrier integrity by tightening tight-junction proteins, including ZO-1, claudin-5, and occludin, and by reducing VEGF-mediated vascular permeability, while simultaneously exerting direct cytotoxic effects on lymphoma cells [90]. Beyond these barrier-level effects, corticosteroids may also influence lymphoma-cell pharmacology relevant to 5-ALA/PpIX imaging. ABC transporters such as ABCG2/BCRP and ABCB1/P-glycoprotein regulate the efflux of multiple endogenous and exogenous substrates, and ABCG2-mediated porphyrin transport is a recognized mechanism limiting intracellular PpIX accumulation after 5-ALA exposure. Pharmacological studies in lymphoid-lineage models indicate that dexamethasone can modulate ABC transporter activity or expression, including MDR1/ABCB1 and BCRP/ABCG2 activity in leukemic cell systems [91,92]. However, the direction and magnitude of glucocorticoid effects appear to be cell-type and context-dependent, and direct PCNSL-specific data linking corticosteroid exposure, ABCG2 modulation, and intraoperative PpIX fluorescence are lacking.

Therefore, poor fluorescence performance in steroid-exposed PCNSL may reflect multiple, partially overlapping mechanisms: reduced barrier permeability, lymphoma cell depletion, and, for 5-ALA/PpIX specifically, a plausible cell-intrinsic reduction in PpIX retention through ABC transporter–mediated efflux. This mechanism should be regarded as hypothesis-generating rather than clinically validated, and direct probe-specific evidence in PCNSL remains limited [93].

## 5. Probe Classes

Having outlined the barrier profiles characteristic of each tumor entity, we now evaluate how major probe classes interact with these constraints (Table 3). For each probe class, we consider not only the dominant transport bottleneck but also major interpretation confounders, including non-neoplastic immune cell uptake, reactive stromal activation, blood or necrotic background, and device-dependent optical filtering. These factors are integrated into the probe-specific discussion because they can decouple observed fluorescence from viable tumor distribution. Importantly, the following subsections are organized not only by mechanism but also by clinical maturity. Metabolic probes such as 5-ALA represent the most clinically established class for IDH-wildtype glioblastoma surgery, whereas fluorescein and ICG-based approaches are clinically used adjuncts with context-dependent and less uniformly validated performance. In contrast, targeted NIR probes, activatable probes, and nanoparticle/carrier-based systems remain largely investigational in CNS tumor surgery; their discussion below should therefore be interpreted as a synthesis of mechanistic rationale, early translational evidence, and potential limitations rather than as evidence of validated clinical performance.

Representative chemical structures and molecular formulas of clinically used small-molecule probes are provided in Supplementary Figure S1; construct-dependent targeted, activatable, and carrier-based probes are summarized in Supplementary Table S1.

### 5.1. Metabolic Probes: 5-Aminolevulinic Acid (5-ALA) and Protoporphyrin IX (PpIX)

5-ALA is an orally administered prodrug that enters the heme biosynthesis pathway as an exogenous precursor and is converted intracellularly to PpIX within mitochondria [94,95]. Cellular uptake is partly mediated by peptide transporters, including peptide transporter 2 (PEPT2, encoded by SLC15A2). PEPT2 has been implicated in 5-ALA transport in astrocytes and the choroid plexus, and higher PEPT2 expression in lower-grade gliomas has been associated with fluorescence positivity [96,97]. One important determinant of tumor-selective fluorescence is metabolic retention of PpIX. Reduced ferrochelatase (FECH) activity, together with limited intracellular iron availability, may impair conversion of PpIX to non-fluorescent heme and thereby favor intracellular PpIX accumulation [95,98]. Additionally, upstream enzymatic flux, regulated by enzymes such as coproporphyrinogen oxidase (CPOX), further modulates PpIX synthesis [95].

In IDH-wildtype glioblastoma and historically defined glioblastoma/GBM cohorts, 5-ALA shows high fluorescence sensitivity in the contrast-enhancing tumor core, where metabolic dysregulation is most evident. In contrast, fluorescence at the infiltrative margin is inconsistent, reflecting reduced tumor cell density and heterogeneous FECH expression [2,99]. In MBTs, 5-ALA fluorescence is variable depending on primary tumor histology. Melanoma and certain adenocarcinomas demonstrate robust PpIX accumulation, whereas other subtypes show limited accumulation [17]. PCNSL represents a particularly challenging setting. In PCNSL, 5-ALA fluorescence is typically weak and inconsistent, likely reflecting both limited delivery following corticosteroid exposure and tumor-specific metabolic characteristics [18]. A cell-intrinsic mechanism may also contribute: corticosteroids could potentially modulate ABC transporter activity in lymphoma cells, thereby reducing PpIX retention, although this remains unvalidated in PCNSL [91,92].

5-ALA is administered orally 3 to 4 h before surgery, permitting adequate time for cellular uptake, enzymatic processing, and PpIX accumulation [100]. Fluorescence intensity peaks within this window and subsequently declines as PpIX undergoes either active efflux or enzymatic conversion to heme. Several mechanisms contribute to diagnostic failure. ABCG2-mediated efflux can export PpIX from tumor cells and has been proposed to reduce intracellular fluorophore concentration in some contexts [29,32]. Optically, photobleaching occurs under prolonged blue-light excitation. Blood within the surgical field absorbs 405 nm excitation light and quenches fluorescence emission [101]. Accordingly, weak or absent fluorescence at the margin should not be interpreted solely as the absence of tumor; it may reflect a combination of low tumor cell density, PpIX efflux or metabolic variability, and optical attenuation by blood or surgical manipulation. The strongest evidence base for 5-ALA remains in IDH-wildtype glioblastoma and historically defined glioblastoma/GBM cohorts, whereas evidence in MBTs is heterogeneous and limited in PCNSL [102,103,104]. Thus, transporter and heme-pathway enzyme expression should be regarded as plausible mechanistic contributors to regional 5-ALA fluorescence variability rather than as validated standalone predictors of intraoperative signal.

### 5.2. Leakage Tracers: Fluorescein Sodium

Fluorescein sodium is a low-molecular-weight (376 Da), hydrophilic xanthene dye that is administered intravenously and accumulates in regions of BTB disruption through passive paracellular diffusion [105,106]. The molecular basis for fluorescein extravasation is tight-junction disassembly, permitting passive passage into the tumor interstitium. Fluorescein neither requires active cellular uptake nor undergoes metabolic conversion; its signal reflects extracellular distribution, making it fundamentally distinct from 5-ALA.

In IDH-wildtype glioblastoma and historically defined glioblastoma/GBM cohorts, fluorescein reliably delineates the contrast-enhancing tumor core, where tight-junction disruption is most pronounced. However, it fails to label infiltrative margins, where the BBB remains intact, representing a consistent limitation [15,21]. In MBTs, fluorescence is typically robust in macrometastases with established neovasculature, but early micrometastases behind an intact BBB frequently escape detection [107]. In PCNSL, performance is highly variable, as PCNSL is particularly susceptible to corticosteroid-induced barrier restoration, abolishing fluorescence signal [108].

Administration strategies vary, with high-dose regimens (20 mg/kg) given at anesthesia induction and low-dose (5 mg/kg) protocols administered intraoperatively [15,23]. The effective imaging window is relatively short, typically spanning minutes to a few hours, governed by intravascular pharmacokinetics, interstitial redistribution and rapid renal clearance. Limitations include non-specific tracer accumulation in peritumoral vasogenic edema and reactive gliotic tissue, rapid interstitial redistribution, and detector saturation at high doses [22], and fluorescence signal attenuation by intraoperative hemorrhage. Because fluorescein signal reflects extracellular leakage rather than tumor cell-specific uptake, fluorescence in edematous brain, reactive gliosis, or inflamed perivascular tissue may not correspond to viable tumor. Conversely, hemorrhage or optical saturation can obscure true signal. These interpretation confounders should be considered alongside barrier status when using fluorescein for margin assessment. Clinical evidence for fluorescein-guided resection is most developed in IDH-wildtype glioblastoma and historically defined glioblastoma/GBM cohorts, whereas evidence in MBTs and PCNSL is more limited and heterogeneous. In PCNSL, corticosteroid-related barrier changes further complicate interpretation.

Recent studies of combined 5-ALA and fluorescein sodium have also evaluated adult-type diffuse gliomas beyond IDH-wildtype glioblastoma, including grade 2 and grade 3 tumors [109,110]. These data suggest that fluorescein signal is closely linked to blood–brain barrier disruption and contrast enhancement rather than to tumor grade alone, reinforcing the need to interpret fluorescein primarily as a barrier-dependent tracer. These reports are relevant to the present framework because they illustrate that fluorescein signal is fundamentally a transport and barrier readout. They do not, however, alter the primary disease scope of this review, which remains IDH-wildtype glioblastoma, MBT, and PCNSL.

### 5.3. Vascular/Near-Infrared (NIR) Dye: Indocyanine Green (ICG)

ICG is an intravenously administered near-infrared (NIR) tricarbocyanine dye that binds approximately 95 to 98% to plasma proteins, predominantly albumin [13,111]. This protein binding increases its effective size, slows diffusion, prolongs intravascular residence, and shifts delivery toward permeability-limited transport. ICG accumulation in tumor tissue is therefore typically delayed and reflects BTB permeability and vascular kinetics rather than cellular uptake.

This delayed pharmacokinetic behavior underpins the second-window ICG (SWIG) approach, in which imaging is performed 18 to 24 h after administration to leverage prolonged retention of albumin-associated signal within permeable tumor microvasculature and interstitium [24,26]. In IDH-wildtype glioblastoma and historically defined glioblastoma/GBM cohorts, SWIG improves the detection of contrast-enhancing tumor but remains limited at infiltrative margins, consistent with spatial heterogeneity in BTB permeability and interstitial transport constraints [13,25]. In MBTs, performance varies with vascular phenotype, particularly between angiogenic and vessel co-option–dominant lesions and associated barrier integrity [112,113]. PCNSL is less well characterized, and barrier restoration following corticosteroid exposure may pose a significant risk of false-negative fluorescence in steroid-exposed patients [28].

ICG supports both immediate angiographic imaging and delayed SWIG protocols. Conventional ICG angiography relies on immediate post-injection imaging to visualize vascular patency and flow dynamics [114]. SWIG requires preoperative dosing and delays intraoperative acquisition, which introduces logistical constraints [25]. As an NIR agent, ICG benefits from reduced autofluorescence and greater tissue penetration depth compared with visible-light probes. However, key limitations include nonspecific fluorescence from edematous brain, hemorrhage, or blood products, inflating background and obscuring margins, dependence on dedicated NIR imaging systems, and false negatives in regions with low permeability (e.g., intact or steroid-restored barrier, vessel co-option–dominant lesions). As with fluorescein, ICG-based signal is not intrinsically tumor cell specific. Blood-pool signal, vascular background, hemorrhage, and edematous tissue can therefore confound interpretation, particularly when delayed retention is used as a surrogate for tumor localization. These limitations should be considered when distinguishing true tumor-associated fluorescence from vascular or inflammatory background. The clinical evidence base is expanding in IDH-wildtype glioblastoma and historically defined glioblastoma/GBM cohorts, more limited in MBTs, and sparse in PCNSL [13,112].

### 5.4. Targeted NIR Probes

Targeted probes conjugate NIR fluorophores to ligands that bind tumor-associated receptors, such as epidermal growth factor receptor (EGFR), integrins (αvβ3), and folate receptor, which are commonly targeted in glioma and metastatic contexts [115,116,117]. For delivery across intact barrier regions, receptor-mediated transcytosis pathways (e.g., LRP1, transferrin receptor, or other transcytosis receptors) may offer a route across relatively intact BBB segments [67,68]. It is important to distinguish this mechanistic rationale from clinical validation. Although receptor expression provides the biological basis for targeted imaging, receptor presence alone does not establish probe suitability in CNS tumor surgery. Effective fluorescence requires both molecular target expression and physical accessibility across the BBB/BTB and tumor ECM. Therefore, targeted NIR probes should currently be regarded as investigational tools in neuro-oncology rather than standard intraoperative adjuncts.

Target receptor expression varies across tumor types, molecular subtypes, and even within individual lesions. EGFR amplification is common in IDH-wildtype glioblastoma but exhibits marked intratumoral heterogeneity, while integrin expression differs substantially across metastatic primaries [118]. Successful targeting requires not only receptor presence but also accessibility, as limited by receptors being buried in dense ECM or behind intact BBB segments. In PCNSL, lymphocyte-specific markers such as CD19 and CD20 represent potential targets, although clinical translation remains at an early stage [119].

Targeted probes typically require hours to days for optimal tumor accumulation, depending on format. Larger constructs exhibit slower extravasation and penetration but longer retention. Smaller peptides extravasate faster but clear quickly [120].

Limitations include false negatives from receptor heterogeneity, immunogenicity with repeated dosing, ECM/barrier impedance to delivery, and potential non-neoplastic uptake depending on probe format. Thus, a positive signal should be interpreted as target-accessible probe accumulation rather than direct proof of viable tumor unless supported by histological validation. Most current evidence remains preclinical or early translational, with limited human neurosurgical experience for select agents [37]. Thus, targeted NIR probes should be interpreted as promising investigational tools rather than clinically validated alternatives to established agents such as 5-ALA, fluorescein, or ICG-based approaches.

### 5.5. Activatable Probes

Activatable probes generate fluorescence only upon interaction with specific enzymatic or microenvironmental triggers, thereby improving tumor-to-background contrast [75,121]. Common activation mechanisms rely on protease-mediated cleavage, including matrix metalloproteinases (MMPs), calpains, and other proteases upregulated in tumor and stromal compartments [38,122]. Some activatable probes are designed for topical application, bypassing vascular delivery constraints entirely [38,122]. The activation therefore depends on local enzyme activity, rather than passive accumulation alone.

Brain tumor–relevant applications of activatable probes have been reported, including topical fluorescent approaches for glioblastoma-relevant detection and experimental protease-activated platforms [38,39]. However, these studies should be interpreted as early translational or preclinical evidence rather than as established clinical validation. Activatable probes are hypothesized to offer advantages for margin assessment when protease activity extends beyond the contrast-enhancing boundary, but this potential advantage remains to be confirmed in larger human neurosurgical cohorts. A central interpretation limitation is enzyme-source ambiguity. Thus, activatable probe signal should be interpreted as a readout of local enzymatic activity, not necessarily as a direct marker of viable tumor cells. MMPs and calpains can be produced by tumor cells but are also expressed by tumor-associated macrophages (TAMs), microglia, and reactive astrocytes [65,123]. In settings with prominent peritumoral inflammation, such as the inflammatory rim surrounding MBTs or the resection edge of IDH-wildtype glioblastoma, immune and stromal activation can yield fluorescence that is not specific for viable tumor, thereby creating a potential false-positive signal.

Activation varies from minutes to hours, depending on enzyme kinetics and probe design [40]. Performance therefore depends on matching the imaging window to local enzymatic activity, which may vary across tumor entities and treatment history. In enzyme-poor regions, incomplete activation can reduce signal and contribute to false-negative margins. Accordingly, the dominant bottleneck for activatable probes is not penetration alone, but the combination of tissue access, activation specificity, and uncertainty regarding the cellular source of the activating enzyme. Key pitfalls therefore include off-target activation by inflammatory or stromal cells, heterogeneity in enzyme expression and activity, and incomplete activation within the operative time window in low-activity regions [41]. Overall, activatable probes have been explored in brain tumor–relevant experimental and early translational settings, including glioblastoma-relevant studies, but they are not standard tools for intraoperative visualization of IDH-wildtype glioblastoma, MBTs, or PCNSL. Their presumed margin-detection advantage should therefore be considered investigational and requires validation in human neurosurgical cohorts [42].

### 5.6. Nanoparticle/Carrier-Based Probes

Nanoparticle-based fluorescent probes encapsulate or conjugate fluorophores within polymeric, liposomal, or inorganic carrier platforms, typically with hydrodynamic diameters of 10 to 200 nm [43,44,45,46,47,48,49,50,52,124,125,126]. The historical rationale for leveraging nanoparticle accumulation in solid tumors has been the enhanced permeability and retention (EPR) effect. Structurally abnormal tumor neovasculature can permit passive nanoparticle extravasation, and limited lymphatic-like clearance can prolong interstitial residence, collectively favoring retention within tumor tissue. However, EPR-mediated delivery is now considered variable and often attenuated in human brain tumors, including IDH-wildtype glioblastoma and brain metastases, with heterogeneous BTB permeability, elevated IFP, and stromal/ECM barriers limiting uniform nanoparticle distribution [44]. In the literature reviewed here, nanoparticle/carrier-based fluorescent probes were not identified as clinically validated intraoperative visualization tools for IDH-wildtype glioblastoma, MBT, or PCNSL. Their inclusion in this review is therefore intended to address a translational design space rather than established neurosurgical practice. The limitations discussed below should be interpreted as potential barriers to applying nanoparticle fluorescence platforms in the brain, extrapolated from preclinical brain tumor delivery studies, transcytosis-based CNS delivery strategies, and broader cancer nanomedicine literature [43]. Accordingly, EPR-based accumulation should not be interpreted as a clinically reliable mechanism for intraoperative brain tumor labeling. In human CNS tumors, nanoparticle delivery remains highly context-dependent, and passive accumulation is best regarded as a design consideration rather than a validated predictor of fluorescence performance.

To address these limitations at the design level, nanoparticle platforms can be engineered to incorporate active targeting ligands or to exploit receptor-mediated transcytosis pathways, including transferrin receptor and LRP1, thereby facilitating transport across BBB/BTB segments that would otherwise exclude particles based on size [50,124,125,126]. TAMs, resident microglia, and surgically recruited phagocytic infiltrates can act as competing cellular sinks for nanoparticle probes, sequestering the probe within non-neoplastic compartments and producing fluorescence that may not correspond to viable tumor cells [52]. Particle size further constrains distribution: larger particles remain perivascular, whereas smaller particles (<50 nm) penetrate more deeply but clear more rapidly, reducing the duration of a usable imaging window [45]. For nanoparticle platforms, therefore, fluorescence distribution should be interpreted as the combined result of tumor access, carrier trafficking, and immune-cell sequestration.

Nanoparticle probes typically require prolonged systemic circulation (hours to days), reflecting slower transendothelial transport and interstitial movement than molecular-scale tracers. Polyethylene glycol (PEG) surface modification (PEGylation) can extend plasma half-life by reducing opsonization and recognition by the mononuclear phagocyte system. However, increased PEG density can reduce cellular uptake, necessitating formulation-specific optimization [46]. The effective imaging window is therefore design-dependent, determined by the interplay among particle size, surface chemistry, BTB transport kinetics, and clearance pathways.

Key limitations include phagocyte-mediated sequestration, variable and often limited EPR contribution in human brain tumors, and size-dependent constraints on interstitial penetration [47,48]. Clinical translation remains limited, and the evidence base is still dominated by preclinical studies [48,49]. Thus, nanoparticle/carrier-based probes should currently be viewed as investigational platforms rather than clinically validated tools for intraoperative visualization of IDH-wildtype glioblastoma, MBT, or PCNSL. Their potential brain-specific limitations include restricted BBB/BTB access, variable EPR contribution, perivascular trapping, phagocyte-mediated sequestration, long circulation requirements, and formulation-dependent clearance.

To avoid conflating clinically validated performance with mechanistic plausibility, we further categorized the principal claims used in the narrative synthesis according to the most direct available evidence. Table 1 summarizes representative claims, the relevant probe or mechanism, the corresponding evidence category, and how each claim is interpreted in this review.

The evidence category reflects the most direct supporting evidence for the specific claim as used in this narrative review. Randomized controlled trial refers to randomized or controlled clinical evidence; prospective cohort refers to prospective human clinical or early-phase studies; retrospective series refers to retrospective clinical case series or institutional experiences; preclinical data only refers to animal, in vitro, ex vivo, engineering, or mechanistic studies without validated human intraoperative performance data. Reviews, guidelines, and meta-analyses were used for contextual interpretation but were not treated as primary evidence categories in this table.

## 6. Goal-Oriented Practical Consideration

**Figure 2:**
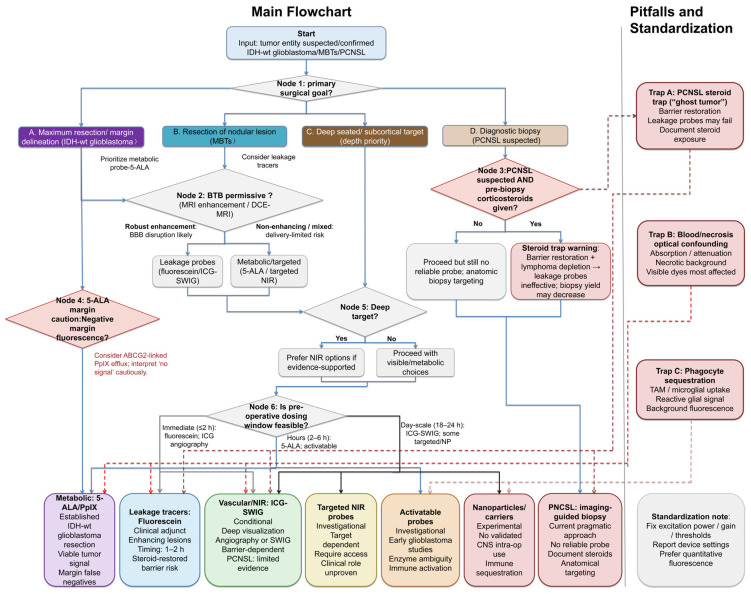
Goal-oriented decision workflow for selecting fluorescence probe classes across IDH-wildtype glioblastoma, metastatic brain tumors (MBTs), and primary central nervous system lymphoma (PCNSL).

Building on the barrier-centric framework and tumor-specific microenvironmental features outlined above, we propose the following goal-oriented, mechanism-informed considerations for intraoperative probe selection and interpretation (Figure 2). Because the level of evidence differs substantially across probe classes and tumor entities, these rules should be interpreted as graded guidance rather than uniform clinical recommendations.

The strength of each rule varies according to the available evidence. In Figure 2 and in the text below, recommendations supported by randomized or prospective clinical evidence are treated as clinically established or conditionally supported, whereas those based on retrospective, case-level, or preclinical evidence are labeled as limited, investigational, or hypothesis-generating. This distinction is particularly important for NIR/SWIG approaches: evidence is more developed for IDH-wildtype glioblastoma and selected MBT settings than for PCNSL, where available SWIG evidence is limited to isolated case-level reports.

Probe choice is organized by surgical goal, barrier status, depth requirement, dosing window, and major interpretation pitfalls. Evidence labels indicate clinical maturity: “Established” denotes routine clinical use in the relevant setting; “Conditional” denotes tumor- and protocol-dependent clinical use; and “Investigational/Experimental” denotes approaches without validated routine neurosurgical application. Preoperative imaging surrogates such as enhancement and permeability imaging are included as operational readouts of the delivery bottleneck but are not intended as validated universal thresholds. ICG/SWIG-based depth guidance should not be extrapolated to PCNSL, where evidence remains sparse and anatomical image-guided biopsy remains the pragmatic approach. Red dashed arrows indicate pitfalls that may decouple fluorescence signal from viable tumor distribution, including corticosteroid-associated barrier restoration, optical confounding, and immune-cell sequestration. The workflow integrates surgical objective, barrier-access proxy, probe mechanism (including potential efflux/metabolic limitations), and practical timing to guide the selection of probe classes: metabolic 5-aminolevulinic acid/protoporphyrin IX (5-ALA/PpIX), leakage tracers, vascular/near-infrared (NIR) agents such as indocyanine green (ICG), targeted or activatable probes, and carrier-based approaches. This workflow is not a validated clinical algorithm; transporter status, ECM features, and advanced barrier phenotyping remain interpretive or investigational decision aids.

Rule 1: Match probe mechanism to surgical goal.

For maximal safe resection in IDH-wildtype glioblastoma, metabolic probes such as 5-ALA remain the most established approach for labeling viable tumor cells, while recognizing the limitations that infiltrative margins may be under-detected [2,127]. In nodular MBTs, leakage-dependent tracers such as fluorescein, and, in selected cases, ICG-SWIG, may facilitate delineation of circumscribed lesions with disrupted BTB [107,112]. In contrast, for PCNSL, where the surgical objective is diagnostic biopsy, no fluorescent probe has demonstrated consistent reliability, particularly following corticosteroid exposure. Preoperative imaging-guided anatomical targeting therefore remains the most pragmatic current approach. These considerations reflect the need to align probe choice with surgical intent and tumor-specific barrier biology highlighted in Section 1 and Section 4. Although isolated case-level SWIG experience has been reported in CNS lymphoma, this evidence remains insufficient to support routine NIR probe selection for PCNSL [128].

Rule 2: Anticipate barrier status before surgery.

Preoperative MRI enhancement patterns, and, where available, DCE-MRI or perfusion imaging, can provide practical but imperfect surrogates for BBB/BTB disruption. Robust enhancement or high permeability may support consideration of leakage-dependent probes, whereas non-enhancing regions, steroid-exposed PCNSL, or suspected vessel co-option should raise concern for delivery-limited false negatives [129]. These imaging surrogates should guide expectations rather than serve as absolute thresholds.

Rule 3: Beware the steroid trap in suspected PCNSL.

In patients with suspected PCNSL, prior corticosteroid exposure can restore barrier integrity and induce tumor cell depletion, substantially reducing the performance of leakage-dependent probes such as fluorescein and ICG. This effect may also lower biopsy diagnostic yield [27]. When clinically feasible, avoidance or deferral of corticosteroid administration may preserve both fluorescence performance and diagnostic tissue yield. Otherwise, reliance on anatomical biopsy targeting remains essential.

Rule 4: Interpret negative 5-ALA margins cautiously.

In 5-ALA-guided resection of IDH-wildtype glioblastoma, absent or weak fluorescence at the tumor edge should not automatically be interpreted as the absence of tumor when navigation, imaging, or histology suggests residual disease. Reduced intracellular PpIX accumulation may reflect low tumor cell density, metabolic variability, optical attenuation, or ABC transporter–mediated efflux [30,32]. At present, however, ABCG2/ABCB1 expression is not routinely used as a clinical decision marker for intraoperative probe selection.

Rule 5: Consider NIR probes for depth in evidence-supported settings.

When resection targets lie beneath the cortical surface or involve deep-seated lesions, NIR probes, including selected ICG-based approaches, may improve subsurface detectability compared with visible-wavelength dyes [117,130]. This recommendation is most applicable to IDH-wildtype glioblastoma and selected metastatic brain tumors; evidence for second-window ICG or other NIR approaches in PCNSL remains very limited. In suspected PCNSL, NIR fluorescence should therefore not replace anatomical image-guided biopsy. Targeted NIR agents remain investigational in neurosurgical oncology and should be considered only in clinical studies or protocol-defined translational use.

Rule 6: Align probe pharmacokinetics with operative workflow.

Probe-specific pharmacokinetics define the practical imaging window. Fluorescein is typically most effective within minutes to a few hours of administration, whereas 5-ALA requires several hours following oral dosing. ICG-SWIG protocols require preoperative administration approximately 18 to 24 h prior to imaging [23,26,100]. Optimal use of fluorescence guidance therefore depends on aligning dosing schedules with the anticipated surgical timeline. For targeted or nanoparticle-based probes, timing windows remain formulation-specific and investigational; they should not be treated as standardized clinical workflows. These timing windows are current workflow considerations rather than patient-specific pharmacokinetic algorithms; individualized timing based on kinetic modeling remains investigational.

Rule 7: Account for immune cell sequestration.

Immune-cell sequestration is an interpretation issue throughout fluorescence-guided surgery, not merely a post hoc limitation. TAMs, microglia, or other reactive non-neoplastic cells can internalize probes, particularly nanoparticles and certain activatable dyes [51,53,123]. This is especially relevant at resection margins, where inflammation is prominent and may lead to overestimation of tumor extent.

Rule 8: Standardize imaging hardware and reporting.

Variability in excitation power, camera gain, and display thresholds complicates signal interpretation [55,58]. Consistent acquisition settings should be maintained, and where possible, quantitative fluorescence measurement should complement visual assessment. Standardized reporting is essential for reliable interpretation and cross-study comparability.

## 7. Limitations of Current Fluorescent Probes

Several limitations discussed above recur across probe classes and therefore deserve explicit synthesis here. As noted throughout the probe-specific sections, a fundamental limitation of current fluorescent probes is limited cellular specificity, particularly in the context of immune and stromal cell uptake. TAMs, which can comprise up to 30–50% of the cellular mass in certain gliomas, can phagocytose nanoparticles, fluorescent dyes, and cellular debris, thereby producing fluorescence signals that may originate from non-neoplastic compartments rather than viable tumor tissue [53,54,65]. Inflammatory and phagocytic cells in peritumoral, treatment-related, or resection-cavity-associated tissue may contribute to false-positive fluorescence. Histology-correlated 5-ALA studies have shown fluorescence in tumor-negative samples containing peritumoral edema, inflammatory-cell infiltration, reactive astrocytes, and CD68-positive macrophages [131,132]. Thus, fluorescence near the surgical cavity should not be assumed to originate exclusively from viable tumor, particularly for probes susceptible to phagocytic uptake or inflammatory activation. This sequestration complicates discrimination between tumor edge, reactive gliosis, and inflammatory infiltrate, and may increase the risk of both over-resection of non-tumor tissue and misinterpretation of fluorescence signal distribution [133].

Optical and device-related factors further decouple observed fluorescence from the underlying probe distribution [56,58]. Without standardized quantification, fluorescence intensity comparisons across patients, anatomical regions, or studies remain difficult to interpret, and reliance on single time-point imaging provides only a static representation of probe distribution and fails to capture dynamic probe kinetics and clearance [57].

5-ALA has the strongest clinical evidence base supporting its use in IDH-wildtype glioblastoma resection [102,134], although much of the older literature used historically defined glioblastoma/GBM cohorts, whereas most other probes remain in preclinical or early-phase clinical evaluation. Direct, head-to-head comparisons between probe classes are rare, and evidence for use in MBTs and PCNSL remains limited. Interpretation of available data is further complicated by heterogeneity of tumor types, prior treatment exposure, and imaging protocols.

This review is subject to limitations inherent to narrative synthesis. Study selection was guided by relevance to the biomechanics-to-fluorescence framework and was not based on a PRISMA-style systematic screening protocol, which introduces potential selection bias. To mitigate this limitation, we provide the search strategy, inclusion priorities, terminology handling, and evidence-categorization approach in Supplementary Materials, Section S1, and we distinguish randomized, prospective, retrospective, and preclinical evidence in Table 3. In addition, the review focuses on three tumor contexts—IDH-wildtype glioblastoma, MBTs, and PCNSL—and does not systematically cover the broader spectrum of adult-type diffuse gliomas or other brain tumors in which fluorescence-guided approaches have been explored, such as meningiomas. Selected references on grade 2–3 adult-type diffuse gliomas are included only when directly relevant to 5-ALA/GlioLAN or fluorescein interpretation.

## 8. Limitation-Driven Future Directions

The future directions below are organized around the principal limitations identified throughout this review: ambiguity of static fluorescence intensity, optical and device-related confounding, immune-cell sequestration, tumor-specific barrier heterogeneity, and limited clinical validation of investigational probe classes. Framing future work around these limitations may help translate the proposed transport-biomechanics framework from a descriptive model into a practical strategy for probe selection and intraoperative interpretation.

First, kinetic fluorescence imaging directly addresses the limitation of static, single-time-point intensity measurements. Static fluorescence cannot reliably distinguish insufficient probe delivery, limited interstitial penetration, poor cellular retention, or rapid clearance. Future strategies should therefore incorporate dynamic wash-in and wash-out metrics rather than relying solely on absolute signal intensity at a single operative time point [135]. Wash-in kinetics may provide a surrogate readout of extravasation, transcytosis, and barrier permeability, whereas wash-out kinetics may reflect retention dynamics shaped by efflux transporters, metabolic processing, and non-neoplastic cellular sequestration. Such kinetic descriptors could help separate biologically distinct failure modes and support more quantitative intraoperative decision-making [136].

This kinetic approach should be evaluated together with preoperative barrier-phenotyping modalities. Prospective studies should test whether permeability imaging, magnetic resonance elastography, diffusion metrics, and intraoperative fluorescence kinetics can predict probe delivery, margin fluorescence, and histological tumor positivity. Such validation will be necessary before the proposed framework can be converted from an interpretive model into a clinically actionable decision-support tool.

Second, multimodal integration is needed to mitigate optical and spatial confounders. Fluorescence intensity can be distorted by blood absorption, necrotic background, tissue scattering, autofluorescence, and device-dependent thresholding, all of which can decouple the observed signal from true tumor distribution. Combining fluorescence with complementary intraoperative modalities, including MRI, ultrasound, and Raman spectroscopy, may help cross-validate spatial localization and improve the interpretation of ambiguous fluorescence patterns [137,138,139]. Similarly, pairing probes with orthogonal mechanisms, such as metabolic probes with leakage-dependent tracers or targeted probes with activatable constructs, could theoretically compensate for single-probe failure modes, although such strategies require prospective validation rather than assumption of additive benefit [16,140].

Third, future probe development and validation should explicitly address cell-of-origin ambiguity. As discussed above, fluorescence signal may arise from tumor cells, endothelial compartments, tumor-associated macrophages, microglia, reactive astrocytes, or surgically recruited inflammatory cells. This is particularly relevant for activatable probes and nanoparticle/carrier-based platforms, where enzymatic activation or phagocytic uptake can generate non-tumor-specific signal. Future studies should, therefore, pair intraoperative fluorescence readouts with spatially resolved histology, immunophenotyping, or molecular validation whenever feasible, so that signal origin can be assigned to neoplastic versus non-neoplastic cellular compartments.

Fourth, patient-specific molecular and barrier phenotyping could address the heterogeneity that limits empiric probe selection. Tumor-specific features such as efflux transporter expression, heme-pathway enzyme activity, vascular phenotype, ECM composition, and steroid-modulated barrier status may influence probe delivery and retention, but these variables are not yet routinely integrated into surgical planning. Molecular profiling could identify tumors at risk for weak 5-ALA/PpIX signal, whereas preoperative barrier phenotyping with dynamic contrast-enhanced MRI or magnetic resonance elastography could estimate permeability and tissue stiffness before surgery [1,135,141]. Such approaches may eventually support patient-specific probe selection rather than reliance on fixed probe algorithms, but their predictive value will require prospective validation against intraoperative fluorescence and histological endpoints.

Finally, investigational probe classes require validation studies designed around clinically meaningful endpoints. Targeted NIR probes, activatable probes, and nanoparticle/carrier-based systems are mechanistically attractive but remain insufficiently validated for routine IDH-wildtype glioblastoma, MBT, or PCNSL surgery. Future studies should therefore report not only tumor-to-background ratios but also margin-level histology, diagnostic yield, false-positive signal from immune or stromal compartments, timing feasibility, workflow burden, and decision impact. Such endpoints would directly address the current gap between mechanistic promise and clinically actionable performance.

## 9. Conclusions

This review proposes a transport- and biomechanics-based framework for interpreting fluorescence probe performance across IDH-wildtype glioblastoma, MBTs, and PCNSL. The main take-home points are:

- Fluorescence signal is not a direct surrogate for tumor presence; it reflects probe delivery, interstitial penetration, cellular retention/clearance, and optical detection constraints.
- Three sequential bottlenecks—BBB/BTB access, ECM penetration, and retention versus clearance—provide a practical framework for comparing probe classes across tumor entities.
- IDH-wildtype glioblastoma, MBTs, and PCNSL differ substantially in barrier integrity, extracellular matrix architecture, cellular organization, and treatment-related confounders, which limits the simple transfer of probe performance from one entity to another.
- 5-ALA/PpIX remains the most clinically established metabolic probe for IDH-wildtype glioblastoma surgery, whereas fluorescein and ICG-based approaches function mainly as context-dependent clinical adjuncts.
- Targeted NIR probes, activatable probes, and nanoparticle/carrier-based systems remain investigational in CNS tumor surgery and should be interpreted as translational opportunities rather than validated clinical alternatives.
- Negative or heterogeneous fluorescence should be interpreted cautiously, particularly in settings of intact or steroid-restored barrier function, ABC transporter–associated efflux, immune-cell sequestration, blood products, necrosis, or device-dependent signal thresholding.
- Future progress will require patient-specific barrier phenotyping, standardized quantitative fluorescence reporting, kinetic imaging, and prospective validation against histology and clinically meaningful surgical endpoints.

## Figures and Tables

**Table 1:** Evidence category for key claims discussed in this narrative review.

Key Claim/Topic	Main Probe or Mechanism	Evidence Category	Representative References from the Manuscript
5-ALA/PpIX is established for IDH-wt glioblastoma surgery	5-ALA/PpIX	Randomized controlled trial	Stummer et al. randomized multicenter phase III trial [2]
Fluorescein highlights enhancing barrier-disrupted glioma tissue	Fluorescein	Prospective cohort	FLUOGLIO multicenter prospective phase II study [15]
Fluorescein evidence remains context-dependent across series	Fluorescein	Retrospective series	Schebesch et al. feasibility series [21]; Neira et al. margin series [22]; Kuppler et al. single-center experience [23]
ICG/SWIG enables timing-dependent NIR glioma visualization	ICG/SWIG	Prospective cohort	Lee et al. near-infrared imaging of gadolinium-enhancing gliomas [24]; Cho et al. second-window ICG in high-grade gliomas [25]
ICG/SWIG is reported for selected brain metastases	ICG/SWIG	Retrospective series	Teng et al. second-window ICG during brain metastasis surgery [26]
5-ALA fluorescence is variable in brain metastases	5-ALA	Retrospective series	Bettag et al. endoscope-assisted 5-ALA visualization in brain metastases [17]
5-ALA evidence in PCNSL remains limited	5-ALA	Retrospective series	Kiesel et al. stereotactic biopsy experience in intracranial lymphomas [18]
Corticosteroids may reduce leakage-probe utility in PCNSL	PCNSL steroid trap	Retrospective series	Scheichel et al. multicenter retrospective PCNSL study [27]; Tosefsky et al. systematic review/meta-analysis [28]
ABC efflux may reduce intracellular PpIX retention	ABC transporter/PpIX efflux	Preclinical data only	Hagiya et al. in vitro transporter study [29]; Ishikawa et al. ABCG2 inhibitor strategy [30]; Bleau et al. ABCG2 network [31]; Chandratre et al. ABCG2 targeting [32]
ECM and IFP may restrict probe penetration	ECM/IFP/stiffness	Preclinical data only	Hyaluronan and stromal resistance studies [33,34]; matrix cross-linking study [35]; tumor mechanics review [36]
Targeted NIR probes require target accessibility	Targeted NIR probes	Prospective cohort	First-in-human cetuximab-IRDye800 glioblastoma study [37]
Activatable probes depend on localized enzyme activity	Activatable probes	Preclinical data only	Topical glioblastoma probe study [38]; activatable cell-penetrating peptide study [39]; protease-activated probe studies [40,41,42]
Nanoparticle probes remain experimental for CNS visualization	Nanoparticles/carriers	Preclinical data only	Nanoparticle delivery and EPR literature [43,44,45,46,47,48,49]; LRP1/transcytosis nanoparticle study [50]; MPS blockade study [51]
Immune-cell uptake may generate non-tumor fluorescence	Immune-cell sequestration	Preclinical data only	TAM imaging review [52]; mononuclear phagocyte system blockade [51]; TAM/microglia review [53]; macrophage polarization study [54]
Blood, necrosis, scattering, autofluorescence, and device settings can distort observed fluorescence.	Optical/measurement confounding	Prospective cohort	Quantitative intraoperative fluorescence imaging [55]; optical tissue characterization [56]; device review [57]; intraoperative optical technologies [58]

Abbreviations: ABCG2, breast cancer resistance protein; 5-ALA, 5-Aminolevulinic Acid; ECM, extracellular matrix; ICG, Indocyanine Green; IFP, interstitial fluid pressure; NIR, Near-infrared; PCNSL, primary central nervous system lymphoma; PpIX, Protoporphyrin IX; SWIG, second-window ICG.

**Table 2:** Candidate Molecular Drivers of Biomechanical and Barrier Phenotypes.

Physical Constraint/Parameter	Candidate Molecular Drivers (Examples)	Representative Readouts/Biomarkers	Implication for Probe Classes
Stiffness/ECM tortuosity	Hyaluronan accumulation [33,34] LOX-mediated Collagen cross-linking [35] Integrin–FAK signaling [76,77]	MR elastography HA/collagen staining LOX expression	Limit penetration of large probes Favor smaller formats Promote rim-biased signal
Barrier permeability (BBB/BTB)	Tight junction remodeling (claudin-5, occludin, ZO-1) [61] VEGF-driven leakiness [20,78] Pericyte coverage Caveola/LRP1 Transcytosis [66]	Contrast enhancement DCE-MRI Tight junction IHC Vascular signatures	Favors leakage tracers when disrupted May limit systemic probes when intact Active/transcytosis strategies may be required
Hydraulic resistance/IFP	VEGF signaling HA water-binding Collagen architecture Drainage constraints [72]	Edema on MRI IFP surrogates ECM composition	Reduces convective delivery Limits deep penetration May shorten effective imaging window
Retention vs clearance	Enzyme activity ABCG2/P-gp Efflux [73] Lysosomal trapping [74]	Transcript/IHC signatures Wash-in/wash-out kinetics	Efflux may cause false negatives Immune or lysosomal uptake may raise background signal Supports kinetic timing optimization

ABCG2, breast cancer resistance protein; BBB, blood–brain barrier; BTB, blood–tumor barrier; DCE, Dynamic contrast-enhanced; ECM, extracellular matrix; FAK, focal adhesion kinase; HA, hyaluronic acid; IFP, interstitial fluid pressure; IHC, immunohistochemistry; LOX, lysyl oxidase; LRP1, lipoprotein receptor-related protein 1; MRI, magnetic resonance imaging; VEGF, vascular endothelial growth factor; ZO-1, zonula occludens-1.

**Table 3:** Probe Catalog.

Probe Class: Example	Metabolic: 5-ALA/PpIX	Leakage Tracer: Fluorescein	Vascular/NIR: ICG (Incl. Delayed)	Targeted NIR Probes	Activatable Probes	Nanoparticles/Carriers
Mechanism	Enzymatic conversion to PpIX; Heme-pathway dependent	Extracellular leakage through disrupted BBB/BTB; No metabolic activation	Albumin-bound vascular/NIR dye; Immediate angiography or delayed SWIG	Ligand/antibody–receptor binding	Signal activated by enzyme/microenvironment	Carrier-based fluorophore delivery; Sometimes ligand-targeted
Dominant bottleneck	Cellular retention; Efflux variability	Barrier-dependent delivery	Barrier-dependent delivery Optical/measurement background	BBB/BTB access ECM and receptor accessibility	Tissue penetration Activation specificity Enzyme-source ambiguity	Extravasation/transcytosis; Immune sequestration
Timing window	3–4 h pre-op; Hours-long window	Intra-op IV Short window	Minutes (vascular) to 12–24 h (delayed)	Hours to days; Format-dependent	Minutes to hours Activation-kinetic dependent	Hours to days; Formulation-dependent
Best-fit scenario (tumor and goal)	IDH-wt glioblastoma resection; Margin support, but variable	Enhancing IDH-wt glioblastoma; Superficial margin support	Vascular mapping; Deeper visualization; Selected enhancing tumors	Potential use in molecularly selected IDH-wt glioblastoma/MBT Requires access	Glioblastoma early data; Investigational margin use; Needs localized enzyme activity	Experimental platform; No validated CNS intra-op use; Brain-imaging design concept
Key strengths	High core contrast; Established workflow	Low cost; Rapid workflow; Useful for enhancing lesions	NIR depth; Surgeon-friendly	Potential specificity; Multiplexable	Higher contrast in principle; Mechanistic insight	Payload flexibility; Multimodal options
Limitations/failure modes	Margin false negatives; Blood absorption; Photobleaching; Transporter/efflux variability	Non-specific leakage; Blood confound; Poor performance with intact BBB/BTB; Steroid-restored barrier risk	Background from blood; Timing critical Barrier-dependent delivery; PCNSL after steroids: false-negative risk	High receptor but low accessibility; Heterogeneity; Off-target binding	Activation in immune/stromal cells; Enzyme-source ambiguity; Incomplete activation in low-activity regions	EPR limited in humans; TAM/microglial uptake; Heterogeneous delivery; Perivascular trapping
Clinical status/evidence hierarchy	Established in IDH-wt glioblastoma; Legacy cohorts included; Variable in MBT; Limited in PCNSL	Clinically used adjunct; Legacy cohorts included; Variable in MBT and PCNSL	Clinical/emerging adjunct; Timing-dependent; Legacy cohorts included; Limited PCNSL data	Investigational/early clinical; Not standard neurosurgical care	Early translational; No standard care; Not established; MBT/PCNSL role	Predominantly preclinical; No validated intra-op CNS use; Validation lacking
Candidate molecular determinants of signal	Ferrochelatase /iron CPOX/enzymes; PEPT2 uptake; ABCG2/P-gp efflux	Tight junction state (claudin-5/occludin/ZO-1); VEGF permeability	Albumin binding; Hydrodynamic radius; BTB permeability; Clearance kinetics	Receptor density; Receptor accessibility; Transcytosis receptors	Tumor vs TAMs enzyme activity source; Microenvironment gradients	Targeting ligand density; Transcytosis engagement; Phagocyte sequestration

Legacy glioblastoma cohorts refer to historical “GBM” studies performed before routine IDH-based molecular classification; these cohorts should not be assumed to represent exclusively WHO CNS5-defined glioblastoma, IDH-wildtype. Abbreviations: ABCG2, breast cancer resistance protein; 5-ALA, 5-aminolevulinic acid; BBB, blood–brain barrier; BTB, blood–tumor barrier; CPOX, coproporphyrinogen oxidase; ECM, extracellular matrix; EPR, enhanced permeability and retention; ICG, Indocyanine green; IDH-wt, IDH-wildtype; IFP, interstitial fluid pressure; MBT, metastatic brain tumors; NIR, near-infrared; PCNSL, primary central nervous system lymphoma; PEPT2, Peptide Transporter 2; PpIX, protoporphyrin IX; TAMs, tumor-associated macrophages; VEGF, vascular endothelial growth factor; ZO-1, zonula occludens-1.

## Data Availability

No new data were created or analyzed in this study. Data sharing is not applicable to this article.

## Supplementary Materials

The following supporting information can be downloaded at: https://www.mdpi.com/article/10.3390/ijms27104495/s1.
