# Supplementary material for: Comparison of Fluorescent Probes for IDH-Wildtype Glioblastoma, Metastatic Brain Tumors, and PCNSL: A Biomechanical Perspective
Source: Int J Mol Sci. 2026 May 17;27(10):4495. doi: 10.3390/ijms27104495 (PMC13207321; doi:10.3390/ijms27104495)
Supplement: Supplementary file 1 [file ijms-27-04495-s001.zip › ijms-4241621-supplementary.pdf]

Supplementary Materials

1. Search Strategy and Evidence Categorization

This work was conducted as a targeted, narrative, mechanistic review intended to develop an interpretive framework for fluorescence probe performance rather than as a formal systematic review or meta-analysis. The primary disease scope was fluorescence-guided surgery in IDH-wildtype glioblastoma, metastatic brain tumors (MBTs), and primary central nervous system lymphoma (PCNSL). We included clinically used probes, including 5-aminolevulinic acid/protoporphyrin IX (5-ALA/PpIX), fluorescein, and indocyanine green (ICG), as well as targeted near-infrared, activatable, and nanoparticle/carrier-based probes when they provided clinically or mechanistically relevant information for brain tumor visualization or CNS delivery.

Targeted searches were performed in PubMed and Web of Science for English-language literature published from January 2005 through December 2025. Searches were supplemented by citation tracking from key clinical trials, translational studies, and mechanistic reviews. Search terms combined the primary disease entities addressed in this review—glioblastoma, IDH-wildtype; metastatic brain tumors/brain metastases; and primary CNS lymphoma/PCNSL—with probe classes and transport-related mechanisms. Probe-related terms included 5-ALA, 5-aminolevulinic acid, GlioLAN, protoporphyrin IX, fluorescein, sodium fluorescein, indocyanine green, ICG, second-window ICG, targeted near-infrared probes, activatable probes, and nanoparticles. Mechanistic terms included blood–brain barrier, blood–tumor barrier, transcytosis, caveolae, LRP1, tight junctions, claudin-5, occludin, hyaluronan, collagen cross-linking, ABCG2, and P-glycoprotein.

For glioblastoma-related literature, the search strategy included both current WHO CNS5 terminology ("glioblastoma, IDH-wildtype") and historical terminology ("glioblastoma" / "GBM") to capture legacy studies that predated routine IDH-based molecular classification. The primary disease scope remained IDH-wildtype glioblastoma, MBTs, and PCNSL. Selected references on other adult-type diffuse gliomas, including grade 2–3 tumors, were included only when they were directly relevant to 5-ALA/GlioLAN or fluorescein sodium interpretation, or when they helped contextualize the limitations of historically defined glioblastoma/GBM cohorts.

Studies were included when they informed at least one of the following domains: (i) molecular determinants of fluorescence signal, such as enzymatic conversion, receptor density and accessibility, efflux transporter activity, or plasma-protein binding; (ii) principal transport bottlenecks, including extravasation/transcytosis, interstitial penetration, retention, and clearance; (iii) timing windows and practical workflow constraints; or (iv) reported performance metrics, interpretation pitfalls, or failure modes. Studies were excluded when they were unrelated to CNS tumors, did not involve fluorescence imaging or probe delivery, focused solely on non-fluorescence imaging or photodynamic therapy without diagnostic fluorescence relevance, or did not contribute to the transport–biomechanics framework. Reviews focused primarily on optical hardware were excluded unless they contributed directly to fluorescence quantification or interpretation.

Because this article is a narrative mechanistic review, a PRISMA-style screening flow, pre-specified numerical yield, and pooled quantitative analysis were not generated. Searches were

iteratively refined to identify clinically and mechanistically relevant evidence, and initial database hit counts were therefore not treated as screening denominators. The final narrative synthesis included 142 references cited in the manuscript.

To make the evidentiary basis of key claims more transparent, representative supporting evidence was categorized into four predefined evidence categories: randomized controlled trial, prospective cohort, retrospective series, and preclinical data only. When a claim was supported by more than one type of evidence, the category shown in Table 3 reflects the most direct evidence supporting the specific statement made in the review. Review articles, guidelines, and meta-analyses were used for contextual interpretation but were not treated as primary evidence categories in Table 3.

To avoid conflating mechanistic plausibility with clinical validation, probe classes were also classified according to clinical readiness. In this review, “clinically established” refers to approaches supported by prospective or randomized clinical evidence and incorporated into routine or guideline-concordant neurosurgical workflows in selected settings, exemplified by 5-ALA/PpIX in IDH-wildtype glioblastoma and historically defined glioblastoma cohorts. “Clinically used adjuncts” refers to probes with human neurosurgical experience but greater dependence on institutional protocols, imaging hardware, dosing strategies, and tumor context, such as fluorescein and ICG-based approaches. “Investigational approaches” refers to targeted NIR probes, activatable probes, and nanoparticle/carrier-based systems for which the rationale is mechanistically compelling but evidence in IDH-wildtype glioblastoma, MBTs, or PCNSL remains primarily preclinical, early-phase, or protocol-specific. Accordingly, investigational approaches are discussed as design concepts and future translational opportunities rather than as clinically validated alternatives to established agents.

2. Probe Structures and Formulas

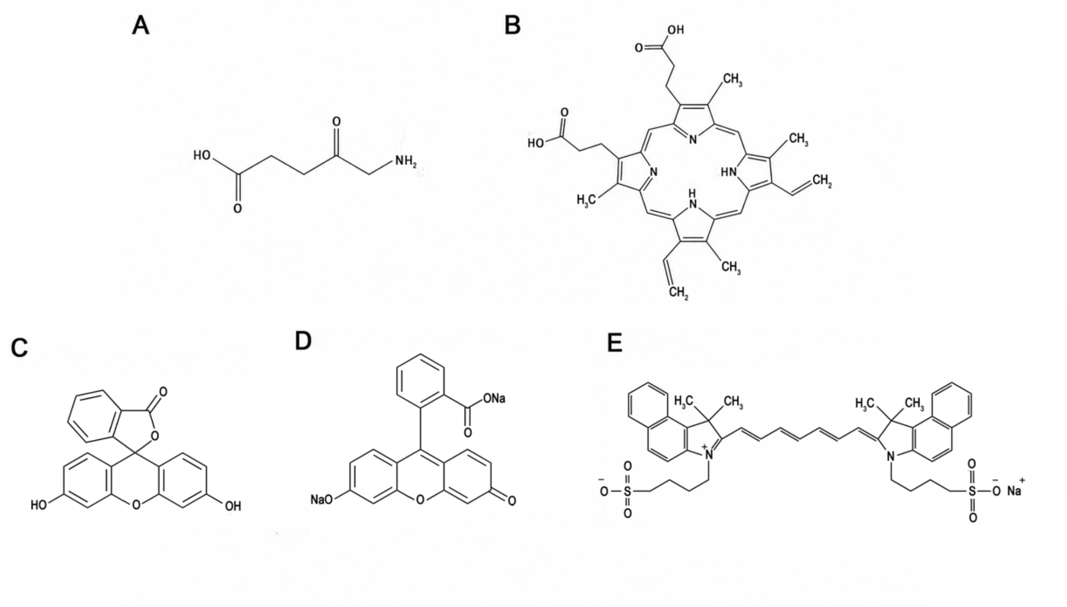

Figure S1. **Representative structural formulas are shown for 5-aminolevulinic acid (5-ALA; A), its fluorescent downstream metabolite protoporphyrin IX (PpIX)(B), fluorescein(C), sodium fluorescein(D), and indocyanine green (ICG)(E)**. These agents represent the principal chemically defined small-molecule probe types discussed in the main text: metabolic labeling (5-ALA/PpIX), leakage-based fluorescence (fluorescein), and near-infrared vascular/permeability-associated imaging (ICG). By contrast, targeted near-infrared probes, activatable probes, and nanoparticle/carrier-based systems do not correspond to a single universal chemical structure because their composition depends on the targeting ligand, fluorophore, linker chemistry, activation mechanism, or carrier platform. These construct-dependent classes are therefore summarized in Table S1 rather than represented by a single formula.

Table S1. Representative formulas and composition notes for reviewed probe classes
Chemically defined small-molecule probes are listed with empirical formulas. For targeted, activatable, and nanoparticle/carrier-based probes, a single formula is not assigned because composition depends on the fluorophore, linker, ligand, quencher, carrier platform, and/or surface chemistry.

Probe / class	Representative formula / composition	Category in review
5-ALA	C5H9NO3	Metabolic probe precursor
PpIX	C34H34N4O4	Endogenous fluorophore
Fluorescein	C20H12O5	Leakage tracer / visible dye
Sodium fluorescein	C20H10Na2O5	Clinical dye formulation
ICG	C43H47N2NaO6S2	Vascular / NIR dye
Targeted NIR probes	Construct-dependent	Investigational targeted probes
Activatable probes	Construct-dependent	Investigational activatable probes
Nanoparticle / carrier-based probes	Formulation-dependent	Investigational carrier systems

Abbreviations: NIR, near-infrared; PpIX, protoporphyrin IX; ICG, indocyanine green.
